# Nanosecond Laser Processing of Titanium in Organic Liquids as a Method for Obtaining Titanium Carbide Coatings

**DOI:** 10.3390/ma18030598

**Published:** 2025-01-28

**Authors:** Rosen Nikov, Nikolay Nedyalkov, Stefan Valkov, Tatyana Koutzarova, Lyubomir Aleksandrov, Genoveva Atanasova, Katarzyna Grochowska

**Affiliations:** 1 Academician Emil Djakov Institute of Electronics, Bulgarian Academy of Sciences, 72 Tsarigradsko Chaussee Blvd., 1784 Sofia, Bulgaria; rosen_nikov@abv.bg (R.N.); stsvalkov@gmail.com (S.V.); tatyana_koutzarova@yahoo.com (T.K.); 2 Department of Mathematics, Informatics and Natural Sciences, Technical University of Gabrovo, 4 H. Dimitar Str., 5300 Gabrovo, Bulgaria; 3 Institute of General and Inorganic Chemistry, Bulgarian Academy of Sciences, Acad. Georgi Bonchev Str. Bld. 11, 1113 Sofia, Bulgaria; lubomir@svr.igic.bas.bg (L.A.); genoveva@svr.igic.bas.bg (G.A.); 4 Center for Plasma and Laser Engineering, The Szewalski Institute of Fluid-Flow Machinery, Polish Academy of Sciences, 14 Fiszera Str., 80-231 Gdańsk, Poland; kgrochowska@imp.gda.pl

**Keywords:** TiC coatings, laser ablation in liquid, liquid paraffin, diesel oil, surface hardness, graphitic phase

## Abstract

This work presents results on nanosecond laser ablation of a titanium (Ti) plate immersed in a liquid medium using the fundamental wavelength (1064 nm) of a nanosecond Nd:YAG laser system. The laser radiation was focused on the target surface as scanning was accomplished by an XY translation stage. The laser processing of the Ti targets took place in two organic liquids—liquid paraffin and diesel oil. The morphology of the structured surfaces and the structure and phase composition of the samples were studied; their dependences on the processing parameters are discussed. With both liquid media used, crack formation on the surface of the laser-treated Ti target was observed. Formation of a titanium carbide (TiC) phase was found whose properties could be tuned by varying the laser irradiation parameters. Raman measurements were utilized to analyze the carbon structure formed in the resulting coatings. The results of surface electron microscopy reveal that the thickness of the resulting coatings reached 20 µm. Some of the obtained coatings demonstrated about three times higher hardness compared to the native Ti sample. The technique proposed can be used in surface modification of materials in view of improving their mechanical properties.

## 1. Introduction

Titanium carbide (TiC) is one of the most interesting materials studied by scientists and technologists due to the combination of desirable properties, such as high hardness, high melting temperature, and chemical and thermal stability [1,2,3]. In most cases, TiC is used as a coating for materials, with these coatings being characterized by high hardness, wear resistance, and thermal conductivity, which make them suitable for a number of industrial applications [4,5,6]. Thus, the main applications of TiC coatings include cutting tools, machine parts, and components exposed to high temperatures and abrasive conditions [7,8].

The most commonly used methods for preparing TiC coatings are physical and chemical vapor deposition (PVD and CVD), magnetron sputtering, pulsed laser deposition (PLD), and carbothermic recovery [5,9,10,11], all of which are excellent for obtaining high-quality and wear-resistant TiC coatings. However, they also have a number of disadvantages, including high cost, toxicity of precursors, and limited layer thickness, as well as difficulty in application to materials with lower heat resistance and complex shapes. CVD usually requires high temperatures (around 800–1100 °C) to decompose the gaseous precursors and initiate the reaction to form the TiC compound. This limits the method’s use on substrates that cannot withstand high temperatures, as many base materials deform, weaken, or undergo structural changes at these temperatures. The equipment for PVD and CVD is complex and expensive, as it involves vacuum systems, reaction chambers, and specialized precursor sources. This makes the processes difficult to implement in smaller-scale production or in applications where cost is a critical factor. CVD often uses toxic, corrosive, and hazardous chemical precursors, such as titanium tetrachloride (TiCl_4_) and methane (CH_4_), which require special safety measures during operation. Improper handling or insufficiently controlled conditions can pose health risks to operators and impact the environment. Both PVD and CVD methods struggle to achieve uniform coating on complex geometric shapes and small openings due to the specific deposition process. On complex surfaces, the layer can become uneven, which limits their applications for parts with intricate geometries.

In recent years, a physical method known as laser ablation of a metal target in an organic solution has begun to gain popularity [12,13,14,15]. Although the method of laser ablation of a target in a liquid medium originally arose as a technique for obtaining complex nano- and microstructures in a liquid (producing colloids), it is increasingly expanding its scope of application. The method of laser ablation in liquid (LAL) is characterized by its simplicity of implementation, environmental friendliness, and flexibility in terms of choice of processing materials (targets) and liquid media. Using LAL, chemical reactions can be induced on the surface, such as oxidation or carburization [13]. The laser pulse can heat the material to high temperatures, causing the surface to react with oxygen or other gases in the environment, creating new compounds, namely oxides or carbides. For example, Soni and co-authors succeeded in synthesizing a TiC coating by laser carburizing on a titanium (Ti) sheet in a controlled methane atmosphere, demonstrating a six-fold increase in the surface hardness of the obtained coating compared to the hardness of a pure Ti substrate [16]. In particular, when irradiating Ti surfaces by laser pulses in an organic liquid, e.g., toluene (which contains hydrocarbons) or ethanol, the laser pulse causes local heating and decomposition of the organic molecules [12] so that carbon is released in the organic liquid. Also, during laser irradiation, the material on the Ti surface is heated to high temperatures, and the laser energy causes a thin layer of the Ti target to melt. The carbon released from the decomposition of the organic liquid diffuses into the molten Ti, where chemical bonds form between the Ti and the carbon, thus forming TiC. After the laser pulse ends, the molten surface rapidly cools, and a TiC phase arises.

Currently, a limited number of studies have appeared in which various experimental parameters have been considered that affect the morphology and chemical composition of surfaces processed during the laser ablation process of a Ti target in organic solutions [12,15]. One such parameter is the type of organic liquid—laser processing in propanol, toluene, n-hexane, n-heptane, and acetone has been reported [12,15]. Laser processing by using different laser systems has been presented, such as a femtosecond laser (laser wavelength of 800 nm, 30 fs, 1 kHz) [15] and a nanosecond laser (fiber laser, 100 ns, 1064 nm, 30 kHz) [12]. In these studies, the influence of the duration of laser processing (number of applied laser pulses) on the morphology and chemical composition of the irradiated Ti surface has also been evaluated. However, information is still lacking on the laser fluence influence on the characteristics of the resulting coatings. For this reason, one of the goals of the present study is to reveal the changes (morphology and chemical composition) that occur in the Ti target surface when the laser fluence is varied during nanosecond laser ablation in an organic liquid. We also expanded the range of organic liquids for LAL to obtain TiC coatings using two relatively environmentally friendly liquids (compared to, e.g., toluene), namely, liquid paraffin and diesel fuel. The choice of these two organic liquids was made because they do not contain oxygen, unlike liquids such as acetone and ethanol, for example; thus the formation of oxide phases (e.g., TiO_2_) is avoided. Further, we present a case of experimental parameters not considered so far in terms of pulse repetition rate, pulse duration, and scanning conditions, and we discuss their effect on the surface properties. Last but not least, the presence of carbon and its crystalline structure (graphitic phases or amorphous carbon) was assessed by measuring the Raman spectra of the obtained coatings.

## 2. Materials and Methods

### 2.1. Laser Ablation Experiments

The laser ablation process of the Ti target in a liquid medium was performed in a way similar to that in [17]. Briefly, the radiation from a nanosecond Nd-YAG laser system (pulse duration 15 ns, repetition rate 10 Hz) was focused by a converging lens onto the surface of a Ti plate immersed in an organic liquid. The Ti target surface was laser-scanned using an XY translation stage. Under the experimental conditions used (spot size and scanning speed), the number of overlapping pulses was 12. These parameters ensured the homogeneous structuring of the surface. Two different organic liquids were used in the ablation process, namely, liquid paraffin (C_n_H_2n+2_, n = 15–40) and diesel oil (C_n_H_2n_, n = 10–22). The choice of these liquids was based on them being less volatile than other organic liquids (e.g., toluene, gasoline, and ethanol) and less harmful to human health. The liquids chosen are also oxygen-free, so the formation of pure carbide phases could be expected. The laser system was used at its fundamental wavelength of 1064 nm. In this study, we chose to process the Ti target by varying the laser fluence in the range of 6.5–10.6 J/cm^2^. The lower value of this interval (6.5 J/cm^2^) fixes the onset of visible modification of the target surface. The laser fluence of 10.6 J/cm^2^ marks the upper end of the range in which effective ablation was observed under these experimental conditions. Further increasing the laser fluence led to an inhomogeneous ablation process with liquid spatter ejection and eventual optical breakdown induced in the liquid.

### 2.2. Characterization Techniques

In most of the cases, three samples obtained at the same experimental conditions were used to obtain statistically reliable results. The organization of the analyses was done in such a way that the same samples were used in different analyses. The morphology of the as-prepared samples was characterized by scanning electron microscopy (SEM, LYRA I XMU Tescan, Brno–Kohoutovice, Czech Republic), operating voltage of 20 kV). The surface roughness of the resulting coatings was evaluated using a 3D optical profiler (Zeta-20, Zeta Instruments, Milpitas, CA, USA). In order to visualize in-depth the resulting coatings and reveal their thickness, SEM analysis of the cross-section of the laser-treated samples was performed. Firstly, the processed samples were immersed in epoxy resin. Then, they were polished using different sandpapers. The final one was P2000. After polishing, the samples were etched in acid solution to develop the microstructure. The crystalline structure and composition of the processed surfaces were explored by an Empyrean diffractometer (PANalytical, London, UK) by glancing (3°) incidence X-ray diffraction (GIXRD) using CuKα radiation. The chemical state of the sample surfaces was studied using X-ray photoelectron spectroscopy (XPS). The XPS studies were performed in an ESCALAB MkII (VGScientific, Manchester, UK) system using AlKα radiation with an energy of 1486.6 eV. The pressure in the chamber was 10^−8^ Pa. The energy calibration was performed by normalizing the C1s line of the adsorbed adventitious hydrocarbons to 284.9 eV. The sample surface was cleaned by Ar^+^ ion sputtering (ion energy of 3 keV, sputtering time of 2 min). The optical properties of both organic liquids used in the laser processing were examined by transmission spectra taken by an HR 4000 spectrometer (Ocean Optics, Ostfildern, Germany). The laser-modified surfaces were also investigated by recording Raman scattering spectra using a Micro-Raman system (InVia Renishaw, New Mills, UK) operated at an excitation wavelength of 514 nm. The microhardness measurements were carried out on EMCO Test DuraScan (Sofia, Bulgaria) equipment. The measurement of the coatings’ hardness was done by a standard technique (ISO 6507-1) [18]. According to the applied procedure, the hardness is evaluated by measurement of an imprint size of the tip of the measurement system. It is performed in the middle of the coating using the geometry of the cross-section. The measurement was not made from the top surface, as in this case, due to the small thickness of the coating, some substrate influence would be introduced. During the experiments, a 20 g load was applied for 10 s.

## 3. Results and Discussion

**Figure 1:**
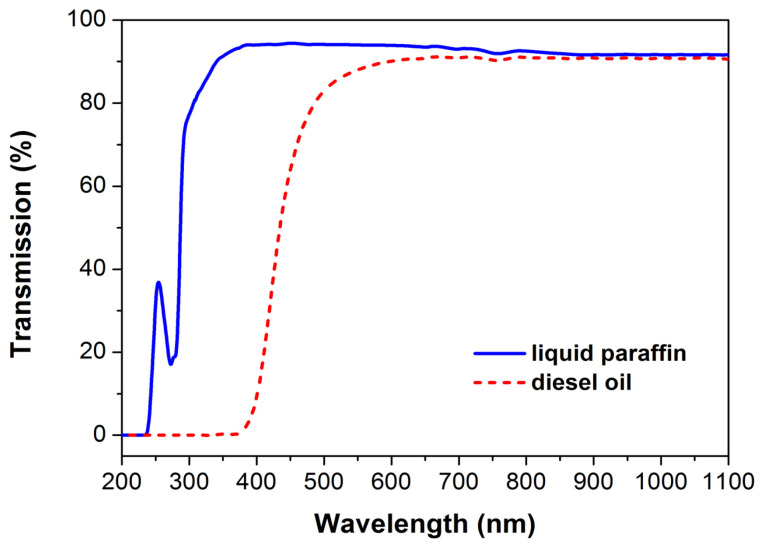
Optical transmission spectra of liquid paraffin and diesel oil.

In the present study, we chose to process the Ti target by the laser’s fundamental wavelength only, since at this wavelength (1064 nm), both liquid paraffin and diesel oil are transparent (Figure 1); therefore, the laser processing of the Ti surface will be more efficient.

### 3.1. Morphology of the Coatings Obtained

**Figure 2:**
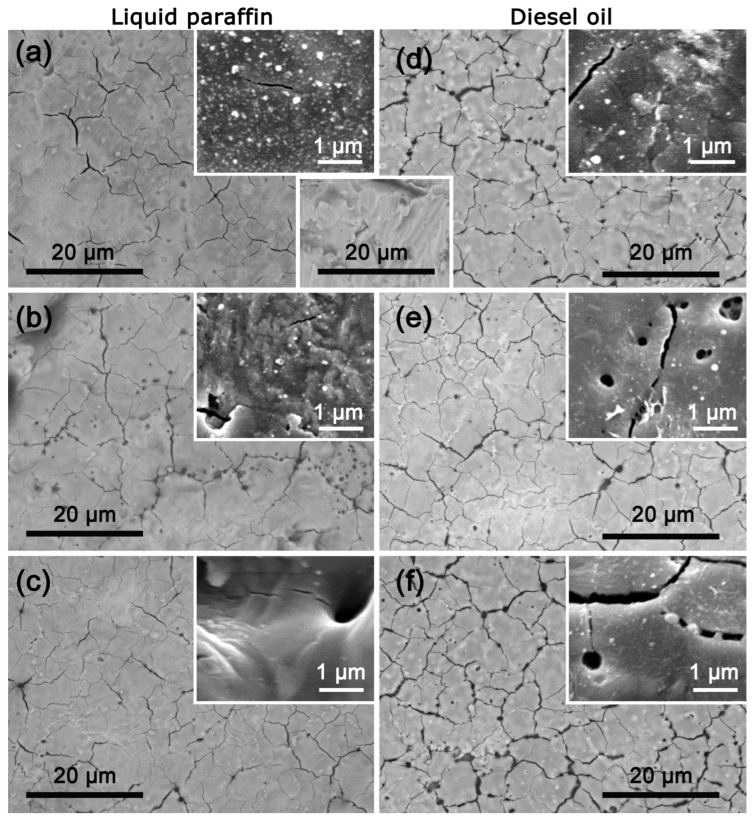
SEM micrographs of the top Ti target surface after laser processing in liquid paraffin (**a**–**c**) and diesel oil (**d**–**f**). The laser fluence applied during the ablation process was 6.5 J/cm^2^ (**a**,**d**), 8.7 J/cm^2^ (**b**,**e**), and 10.6 J/cm^2^ (**c**,**f**). The corresponding SEM images with higher resolution are also inserted. The inset between (**a**) and (**d**) is an SEM image of the Ti surface before laser treatment.

The first series of experiments on laser processing of a Ti target was performed in the liquid paraffin environment. As mentioned above, a range of laser fluence values was established where effective ablation of the Ti target was realized. Therefore, the laser processing of the Ti surface was carried out using the two limit values of this range (6.5 J/cm^2^ and 10.6 J/cm^2^), as well as an intermediate value, namely 8.7 J/cm^2^. SEM micrographs of the top Ti target surface after laser irradiation in liquid paraffin at laser fluences of 6.5 J/cm^2^, 8.7 J/cm^2^, and 10.6 J/cm^2^ are presented in Figure 2a–c, respectively. In each of these images, corresponding higher-resolution SEM images are inserted for a more detailed characterization of the surface structure of the resulting coatings. The native surface morphology of the Ti target before laser treatment is also presented in Figure 2 (inset between (a) and (d)). The laser processing leads to a surface where a crystallite-defined morphology is not present, in contrast with the native sample. Furthermore, the laser irradiation leads to the formation of cracks on the target surface in all three cases (Figure 2a–c). As a trend, it can be noted that the density of the cracks increases as the laser fluence is increased. Moreover, in addition to cracks, the appearance of microholes or voids on the target surface can be seen, the most noticeable ones being in the case of laser processing with a fluence of 8.7 J/cm^2^ (Figure 2b). The formation of cracks during laser processing of metal surfaces is often observed, especially when short and ultrashort laser pulses are used [19,20]. Several factors related to the intensity of the ablation process and the specific physical properties of the material can contribute to the appearance and propagation of cracks on the target surface. The first factor that leads to the appearance of cracks is the thermal stress [21]. Laser ablation is a process that uses high-energy laser radiation to vaporize small amounts of material from the surface. The intense, rapid heating of the surface layer causes a significant temperature gradient. This rapid heating and subsequent cooling lead to uneven expansion and contraction, creating considerable thermal stress. As a result, cracks may form if the material’s strength is exceeded.

Another factor in the appearance of cracks is the creation of shock waves during the LAL process. It is known that cavitation bubbles are formed during the LAL process, especially with high-energy pulses [22]. These bubbles expand and quickly collapse, creating strong shock waves in close proximity to the target; these lead to significant mechanical stress on the target surface, which can result in cracks. The rapid expansion and collapse of cavitation bubbles create fluctuating pressure on the target’s surface, leading to additional mechanical stresses that promote cracking. On the other hand, when these cavitation bubbles collapse or detach from the target surface, microholes are formed on the surface [23]. The aforementioned processes contribute to the formation of coatings with cracks and small holes (below 1 μm in diameter), as observed in Figure 2. A close look at the higher-resolution SEM images reveals the presence of bright nanoscale features, which are most likely redeposited material ejected during the ablation process (Figure 2a,b). The laser processing with intermediate and high laser fluence leads to the formation of relief structures, as seen in the higher-resolution SEM images in Figure 2b,c, respectively.

The presence of cracks and microholes is also observed during laser treatment in diesel oil at the same laser fluences—6.5 J/cm^2^, 8.7 J/cm^2^, and 10.6 J/cm^2^ (Figure 2d–f). It can even be noted that processing in diesel oil leads to both a greater crack density and a greater crack width, especially in the case of using 10.6 J/cm^2^ (Figure 2f). There are several possible reasons for the appearance of more and larger cracks on the target surface at higher laser fluence. Increased laser fluence means more energy is delivered to the target surface; hence, higher local temperature and, eventually, greater thermal stress is generated. In addition, at higher laser fluences, the energy transferred to the surrounding liquid generates larger, more energetic cavitation bubbles. When these bubbles collapse near the target surface, they create stronger shockwaves that can propagate through the target surface. This repeated high-pressure impact can induce mechanical stresses and exacerbate crack formation or deepen pre-existing micro-cracks [24]. Higher-resolution SEM images reveal that laser treatment in diesel oil also results in the redeposition of material in the form of nanoscale structures on the resulting coatings (Figure 2d–f).

**Figure 3:**
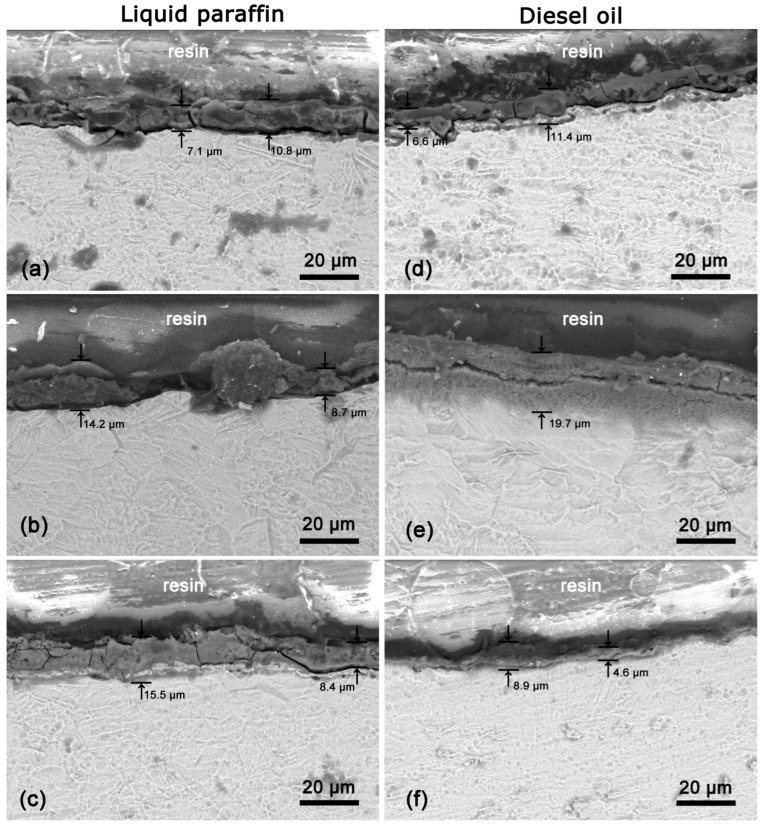
Cross-section SEM micrographs of Ti targets after laser processing in liquid paraffin (**a**–**c**), and diesel oil (**d**–**f**). The laser fluence applied during the ablation process was 6.5 J/cm^2^ (**a**,**d**), 8.7 J/cm^2^ (**b**,**e**), and 10.6 J/cm^2^ (**c**,**f**).

To visualize in-depth the coatings and to estimate their thickness, cross-sections of the Ti targets were made. SEM images of cross-sections of coatings obtained by laser processing of Ti targets immersed in liquid paraffin and diesel oil are shown in Figure 3. The laser processing using 6.5 J/cm^2^ of a Ti target in liquid paraffin results in the formation of coatings with thicknesses ranging from 7.1 to 10.8 µm (Figure 3a). Increasing the laser fluence value to 8.7 J/cm^2^ leads to an increase in the two limit values of the coating thickness, namely 8.7 and 14.2 µm (Figure 3b). It should also be noted that the coating in Figure 3b appears more inhomogeneous in thickness compared to that obtained with low laser fluence (Figure 3a). The laser processing with the highest fluence leads to the formation of coating with a thickness ranging from 8.4 to 15.5 µm (Figure 3c), which in terms of values is close to those of the coating obtained at intermediate laser fluence (Figure 3b).

On the other hand, in the case of laser processing in diesel oil, no clear dependence of the coating thickness on the applied laser fluence is observed. The processing with low laser fluence leads to the formation of a coating with a thickness varying in the range of 6.6–11.4 µm (Figure 3d). A relatively homogeneous coating with a thickness of about 19.7 µm was observed during laser processing with an intermediate (8.7 J/cm^2^) laser fluence (Figure 3e). In this case, a longitudinal crack dividing the coating approximately in the middle is also observed (Figure 3e). The reason for its appearance may lie in the sample preparation for this type of analysis, namely, cutting and subsequent polishing of the target. The laser processing of a Ti target with the highest fluence, in turn, generally leads to the formation of a thinner coating with a thickness ranging between 4.6 and 8.9 μm (Figure 3f).

In the case of processing the Ti target in liquid paraffin, a larger variation of the coating thickness is observed, especially when using intermediate (8.7 J/cm^2^) fluence (Figure 3b). In both cases of the liquids (diesel oil and liquid paraffin), the same laser scanning conditions were used. The observed thickness variation in the case of ablation in paraffin could be related to a change in the pulse overlapping due to a change in the laser spot size on the surface. This change is related to the different refractive indices of the liquids. Furthermore, the liquid paraffin has a higher viscosity, which limits the removal efficiency of the ablated material from the ablation spot. This will also affect the spatial distribution of the laser irradiation and the absorbed energy by the target.

**Figure 4:**
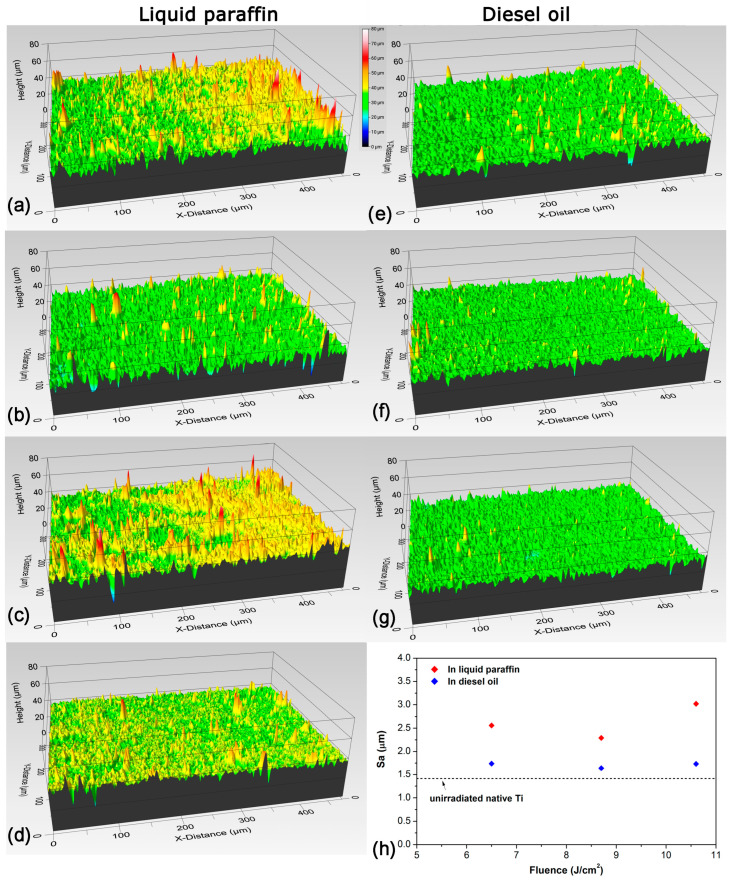
Optical profilometer 3-D images of Ti targets after laser processing in liquid paraffin (**a**–**c**), and diesel oil (**e**–**g**). The laser fluence applied during the ablation process was 6.5 J/cm^2^ (**a**,**e**), 8.7 J/cm^2^ (**b**,**f**), and 10.6 J/cm^2^ (**c**,**g**). The profile of the native Ti target before laser processing is shown in (**d**). The dependence of the roughness parameter Sa of the coatings on the applied laser fluence is presented in (**h**).

From the perspective of achieving a more detailed study of the surface structure and roughness of the obtained coatings, measurements were performed with an optical profilometer. Figure 4 presents the three-dimensional (3-D) profiles of the coatings obtained by laser processing in liquid paraffin and diesel oil using different laser fluences. An image of the surface profile of the native Ti target before laser processing is presented in Figure 4d. From the presented 3-D profiles of the coatings, it can be seen that laser processing of Ti in liquid paraffin with low (Figure 4a) and high fluence (Figure 4c) leads to higher surface roughness compared to processing with intermediate fluence (Figure 4b). In the case of laser processing in diesel oil, it is seen that there is no significant change in the roughness of the coatings obtained using 6.5 J/cm^2^, 8.7 J/cm^2^, and 10.6 J/cm^2^ (Figure 4e,f, and d, respectively). It can be noticed that coatings obtained by laser processing in diesel oil have less roughness; that is, they are smoother than coatings obtained by processing in liquid paraffin (Figure 4). In order to confirm this statistically, the roughness parameter Sa was measured on the resulting coatings, which expresses in absolute value the difference in the height of each point compared to the arithmetical mean of the surface.

The dependence of the roughness parameter Sa on the laser fluence by which Ti surfaces were processed is shown in Figure 4h. The values of the Sa parameter of the coatings obtained by laser processing in diesel oil vary in the range of 1.639–1.739 µm, while for processing in liquid paraffin, the range is from 2.291 to 3.019 µm. The value of the Sa parameter for an untreated Ti surface is 1.414 µm and is also shown for comparison in Figure 4h. The results clearly show that the coatings obtained by laser processing in diesel oil are smoother than those obtained by laser processing in liquid paraffin. It should be noted that in both treatments in liquid paraffin and diesel oil, the coatings obtained with intermediate fluence have the smallest Sa parameter; i.e., they are the smoothest.

In order to compare the presented results, we refer to Ref. [12], where TiC is formed by laser ablation in liquids at experimental conditions closest to those presented here. However, in the aforementioned study, the laser treatment was performed using a significantly higher laser pulse repletion rate (×3000), about an order of magnitude longer laser pulses, and in different liquid media. The authors of [12] also performed multiple scanning of the samples. A comparison of the characteristics of the coatings obtained shows that under the conditions used in [12], the surface film of TiC was more homogeneous and contained fewer cracks. One could assume that the film characteristics could be improved by a multiple-sample scanning. However, we believe that using pulses with a duration of tens of nanoseconds and a low repetition rate could ensure unique conditions for high-temperature gradients that are crucial for coating characteristics such as hardness and wear resistance. The short laser pulses may also produce films of sub-micrometer thickness for application in, e.g., MEMS.

### 3.2. Microstructure and Composition of Obtained Coatings

#### 3.2.1. XRD Analysis

**Figure 5:**
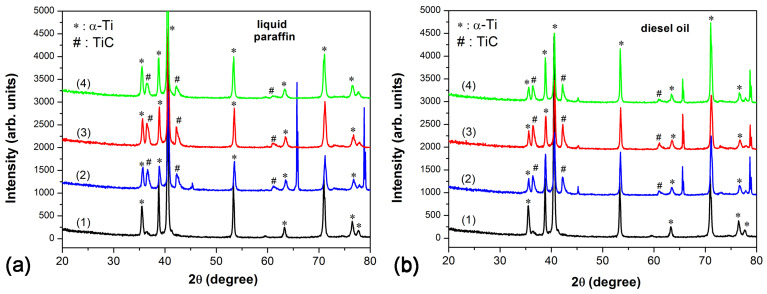
XRD patterns of Ti target surfaces before and after laser irradiation in liquid paraffin (**a**) and diesel oil (**b**) at different laser fluences (F): (1) without laser processing, (2) F = 6.5 J/cm^2^, (3) F = 8.7 J/cm^2^, and (4) F = 10.6 J/cm^2^.

XRD analyses were performed in order to reveal the changes occurring in the crystal structure and composition of the Ti target surface after laser processing. The XRD patterns of the surfaces before and after laser treatment using different laser fluences in both liquid paraffin and diesel fuel are presented in Figure 5a and Figure 5b, respectively. In all diffractograms of the samples before and after laser treatment, diffraction peaks are identified corresponding to the crystallographic planes (100), (002), (101), (102), (110), (103), (200), and (112) of hexagonal structure of α-Ti (ICSD 98-005-2522). The laser processing of the Ti target at all three laser fluences (6.5 J/cm^2^, 8.7 J/cm^2^, and 10.6 J/cm^2^) in both liquids used (spectra (2), (3), and (4) in Figure 5a,b), in turn, leads to the appearance of diffraction peaks located at 36.41°, 42.19°, and 61.11° corresponding to (100), (112), and (200) of the cubic structure of TiC (ICSD 98-018-0598).

The mechanism of TiC formation during laser irradiation of a Ti target in an organic liquid can be summarized as follows: the interaction of high-energy laser pulses with a Ti target creates a plasma plume, which, upon contact with the liquid, leads to the decomposition of the organic molecule of the liquid and the release of carbon. Also, as a result of the nanosecond laser ablation, a thin layer of the Ti target surface melts, allowing the released carbon to diffuse into the molten Ti, forming a TiC phase. Analyzing the XRD results, one can notice that increasing the laser fluence from 6.5 J/cm^2^ to 8.7 J/cm^2^ leads to an increase in the TiC peak intensity, most notably those at 36.41° and 42.19°, for both processing media used—liquid paraffin and diesel oil (spectra 2 and 3 in Figure 5). More intense diffraction peaks could indicate a greater amount of TiC phase formed during the higher-fluence laser processing [12]. It is interesting that further increasing the laser fluence to 10.6 J/cm^2^ leads to a significant decrease in the diffraction peaks of the TiC phase in the formed coatings (spectrum 4 in Figure 5a,b). To understand the reasons leading to the above results, a detailed examination and understanding of the LAL process is required. Although the aims of the present work do not include such a detailed study, here we will attempt to explain very briefly the laser fluence effect on the formation of TiC in the coatings. As the laser fluence increases, the heat-affected zone increases; hence, more material is heated, vaporized, and ionized, resulting in the formation of a larger plasma plume. The increased energy also increases the temperature and pressure of the plasma plume, which enhances its expansion. The larger the plasma plume, the larger the volume of liquid it contacts, which means that more organic molecules will decompose, and a larger amount of carbon will be released. More carbon in the vicinity of the target–liquid interface means a greater probability of a TiC structure formation in the resulting coating. On the other hand, as the laser fluence increases, the amount of material ejected from the target into the liquid also increases. At higher concentrations, this laser ablation product, in the form of nanoparticles, microparticles, and clusters, can shield the target (absorption in and scattering of the particles), thus reducing the efficiency of the ablation process. Liquids of higher viscosity, such as liquid paraffin, prevent the rapid dispersion of the particles formed in the liquid, leading to reduced ablation efficiency and, hence, a reduction in the TiC content in the obtained coating. In addition to the peaks of α-Ti and TiC in the XRD spectra of laser-treated Ti surfaces in diesel oil (spectra 2, 3, and 4 in Figure 5b) and the XRD spectrum of the Ti target processed with 6.5 J/cm^2^ in liquid paraffin (spectrum 2 in Figure 5a), clearly pronounced peaks located at 65.59° and 78.69° are observed. These peaks can be attributed to the (211) and (220) crystal planes of the cubic β-Ti phase (JCPDS file 44-1288). During laser irradiation, the Ti target surface temperature could increase significantly, exceeding the α-Ti to β-Ti phase transition temperature (~882 °C for pure Ti) [25]. Laser processing creates high-temperature gradients that lead to a partial phase transition from α-Ti to β-Ti. The lower temperature zones may retain the α-Ti phase while the hotter regions transition to the β-Ti phase. Studies on Ti alloys such as Ti-6Al-4V have shown that after laser processing, zones where α-Ti and β-Ti phases coexist are often observed, depending on the local heating and cooling conditions [26]. It should be noted that the diffraction peaks at 65.59° and 78.69° observed in Figure 5 are shifted relative to those of pure β-Ti (69.6° and 82.45°). The reason for this may be the formation of an alloy of α- and β-Ti, which changes the microstructure of the material, and the positions of the diffraction peaks are shifted relative to those of the pure Ti phases. Ti alloys often contain both α and β phases [27]. In diffraction analysis, peaks from the two phases may overlap, or additional peaks may appear as a result of interaction between the phases.

#### 3.2.2. X-Ray Photoelectron Spectroscopy

**Figure 6:**
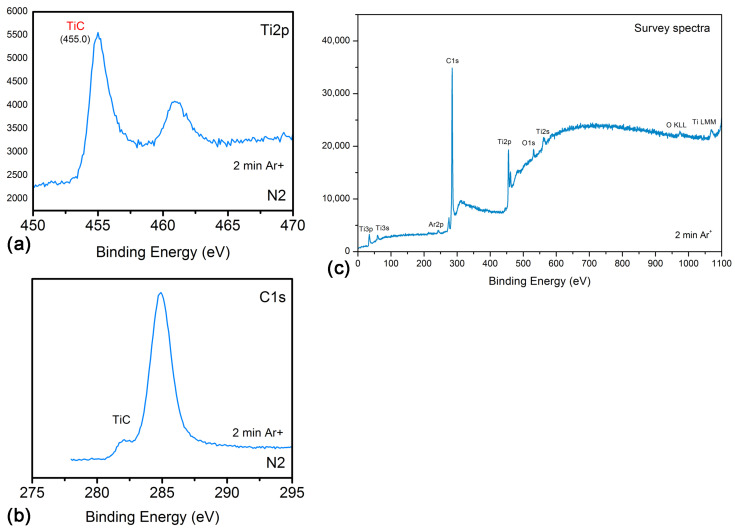
(**a**) Ti2p and (**b**) C1s XPS spectra of Ti target surface after laser irradiation in liquid paraffin. The laser fluence used for surface processing was 8.7 J/cm^2^. (**c**) The survey XPS spectrum is also presented.

Additional analyses of the composition and electronic structure of the coatings obtained were carried out by X-ray photoelectron spectroscopy (XPS). Since the results of the XRD analysis did not show a significant difference in the composition of the coatings obtained by laser processing in liquid paraffin and diesel oil, we continued further research for the case of laser processing in liquid paraffin only. In order to remove surface contamination resulting from exposure to air before placing the sample into the XPS system, the coatings were Ar^+^ ion etched. Figure 6 shows the XPS spectra of a Ti surface laser irradiated by a fluence of 8.7 J/cm^2^ in liquid paraffin. The two peaks in Figure 6a centered around 455 eV and 461 eV correspond respectively to the main line (Ti2p_3/2_) and second line (Ti2p_1/2_) of Ti in TiC [28]. On the other hand, two peaks at 282.1 eV and 284.9 eV are observed in the C1s spectrum (Figure 6b). Typically, the carbon line in TiC is located around 281.5–282.5 eV. This is a characteristic of carbon bonded to a metal (carbide compounds). The presence of a peak around 284.9 eV in the C1s spectrum is due to adsorbed adventitious hydrocarbons.

#### 3.2.3. Raman Spectroscopy

**Figure 7:**
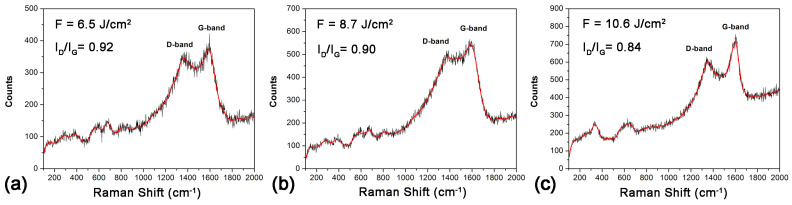
Raman spectra of Ti target surfaces after laser irradiation in liquid paraffin at different laser fluences: (**a**) F = 6.5 J/cm^2^, (**b**) F = 8.7 J/cm^2^, and (**c**) F = 10.6 J/cm^2^.

Measurements of the Raman scattering spectra of the laser-processed samples were carried out in order to thoroughly study the microstructure of the coatings obtained. Figure 7 presents the Raman spectra of the samples obtained by laser processing in liquid paraffin using three laser fluences—6.5 J/cm^2^ (a), 8.7 J/cm^2^ (b), and 10.6 J/cm^2^ (c). In all three Raman spectra, one can see two broad, intense peaks centered around 1590 cm^−1^ and 1360 cm^−1^ corresponding to the G-band and D-band, respectively, indicating the presence of free carbon phases in the coatings [12]. The G-band arises from the E_2g_ vibrational mode, which involves the in-plane stretching motion of sp^2^-bonded carbon atoms. This mode is a signature of a crystalline-graphitic structure and an indicator of the presence of ordered graphitic domains. In turn, the D-band, or defect band, is activated by a process called double-resonance Raman scattering. It involves defects or disorder in the carbon lattice, such as edges, vacancies, or functional groups that break the perfect symmetry of the sp^2^-hybridized network. The ratio of the D-band-to-G-band intensity (I_D_/I_G_) is indicative of the ratio of amorphous carbon to the ordered graphite. Observing Figure 7, it can be concluded that by increasing the laser fluence, the ratio I_D_/I_G_ decreases, pointing to an increase in the portion of the ordered graphitic phase relative to that of amorphous carbon in the resulting coating.

The presence of peaks between 200 cm^−1^ and 700 cm^−1^ in the three Raman spectra in Figure 7 confirms the results of the XRD analysis for the presence of a TiC compound in the coatings obtained. The peaks located at 286, 378, 574, and 678 cm^−1^ (Figure 7a,b), as well as those at 335 and 640 cm^−1^ (Figure 7c), are in good agreement with the characteristic Raman peaks of non-stoichiometric TiC_x_ (x < 1) reported by other authors [29]. However, it should be noted that these peaks, arising from vibrational modes of non-stoichiometric TiC_x_, are of relatively weak intensity compared to those observed by other authors [30], which means that the content of TiC_x_ in the obtained coatings is very small compared to the share of stoichiometric TiC.

### 3.3. Hardness Analysis

To determine the hardness of the resulting coatings, a measurement was made on their cross-section. The measurements show that the hardness of the coating obtained by laser processing of a Ti target in diesel oil is 596 HV, while the measured hardness of the unirradiated Ti target is 194 HV. Thus, the laser surface treatment leads to an approximately threefold increase in the hardness of the tested material. The reason for the increased hardness of the Ti sample surface after laser processing is that a new phase has formed, namely TiC.

## 4. Conclusions

This work describes an environmentally friendly, effective, and easy-to-implement method for preparing TiC coatings based on modifying the surfaces of solid targets using the LAL method. Specifically, in the present study, nanosecond laser ablation of a Ti target in two organic liquids, namely liquid paraffin and diesel oil, was performed to form the TiC coatings. The laser fluence effect on the morphology and composition of the resulting coatings was investigated. For this purpose, laser processing of Ti samples was performed using three fluences, namely 6.5, 8.7, and 10.6 J/cm^2^. In general, as the laser fluence is increased, more defects (cracks and microholes) are observed in the coatings in both ablation media, namely, liquid paraffin and diesel oil. Optical profilometer measurements reveal that coatings obtained by laser processing in diesel oil are smoother than those obtained by processing in liquid paraffin. The XRD analyses performed show that the TiC phase is formed at all three laser fluences used for both liquid media. The XRD spectra indicate that the highest content of the TiC phase in the coatings is contained in the samples obtained by intermediate fluence processing. The XPS analysis confirms the presence of a TiC phase in the coatings and also suggests the formation of carbon structures. Raman measurements reveal that the coatings contain both TiC_x_ (x < 1) phases as well as carbon phases in the form of ordered graphite and amorphous carbon. An almost threefold increase in the surface hardness of the Ti sample after laser treatment in diesel oil is demonstrated. The proposed method could be applied as an efficient alternative to electron and ion technologies (which require a vacuum) for surface modification. The formed TiC coatings can be used to improve the performance of the materials with applications in the aircraft, automotive, and material-processing industries. Future efforts will be directed towards improving the technology for creating coatings with minimal crack formation, homogeneity of the coating thickness, and increasing their hardness, as well as a comprehensive study of the influence of process parameters on the properties of the coatings.

## Data Availability

The original contributions presented in this study are included in the article. Further inquiries can be directed to the corresponding author.
